# Epstein-Barr virus infection-induced inflammasome activation in human monocytes

**DOI:** 10.1371/journal.pone.0175053

**Published:** 2017-04-03

**Authors:** Yuka Torii, Jun-ichi Kawada, Takayuki Murata, Hironori Yoshiyama, Hiroshi Kimura, Yoshinori Ito

**Affiliations:** 1 Department of Pediatrics, Nagoya University Graduate School of Medicine, Nagoya, Japan; 2 Department of Virology, Nagoya University Graduate School of Medicine, Nagoya, Japan; 3 Department of Microbiology, Shimane University Faculty of Medicine, Izumo, Shimane; University of Nebraska-Lincoln, UNITED STATES

## Abstract

Inflammasomes are cytoplasmic sensors that regulate the activity of caspase-1 and the secretion of interleukin-1β (IL-1β) or interleukin-18 (IL-18) in response to foreign molecules, including viral pathogens. They are considered to be an important link between the innate and adaptive immune responses. However, the mechanism by which inflammasome activation occurs during primary Epstein-Barr virus (EBV) infection remains unknown. Human B lymphocytes and epithelial cells are major targets of EBV, although it can also infect a variety of other cell types. In this study, we found that EBV could infect primary human monocytes and the monocyte cell line, THP-1, inducing inflammasome activation. We incubated cell-free EBV with THP-1 cells or primary human monocytes, then confirmed EBV infection using confocal microscopy and flow cytometry. Lytic and latent EBV genes were detected by real-time RT-PCR in EBV-infected monocytes. EBV infection of THP-1 cells and primary human monocytes induced caspase-dependent IL-1β production, while EBV infection of B-cell or T-cell lines did not induce IL-1β production. To identify the sensor molecule responsible for inflammasome activation during EBV infection, we examined the mRNA and the protein levels of NLR family pyrin domain-containing 3 (NLRP3), absent in melanoma 2 (AIM2), and interferon-inducible protein 16 (IFI16). Increased AIM2 levels were observed in EBV-infected THP-1 cells and primary human monocytes, whereas levels of IFI16 and NLRP3 did not show remarkable change. Furthermore, knockdown of AIM2 by small interfering RNA attenuated caspase-1 activation. Taken together, our results suggest that EBV infection of human monocytes induces caspase-1-dependent IL-1β production, and that AIM2, acting as an inflammasome, is involved in this response.

## Introduction

Epstein-Barr virus (EBV) is one of the most prevalent human viruses in the world. It is estimated that over 90% of adults in developing countries are EBV seropositive. Many children do not experience symptoms during EBV infection, but adults, adolescents, and some older children may present with some or all of the typical signs and symptoms of infectious mononucleosis: fever, lymphadenopathy, pharyngitis, and splenomegaly. When EBV enters the body after infection, it usually proliferates in oropharyngeal epithelium prior to entering the bloodstream. Following viremic dissemination, EBV remains latent in B lymphocytes throughout life.

There have been several reports that have shown EBV infection of monocytes [1,2]. Innate immune cells such as monocytes and macrophages may play a role in the primary infection or reactivation of EBV, but the mechanism of such involvement is unclear. EBV infection is mainly controlled by adaptive immunity, and little is known about inflammasome activation in response to primary EBV infection. A recent study showed elevated serum IL-18 levels in patients with acute EBV infection [3], which may indicate an innate immune response to primary EBV infection. Furthermore, Ansari et al. demonstrated inflammasome activation in B lymphocytes and epithelial cells during EBV latency [4]. Inflammasome activation in monocytes during primary EBV infection, however, has not been demonstrated.

Inflammasomes, the cytoplasmic sensors that regulate caspase-1 activity and IL-1β or IL-18 secretion induced by foreign molecules (including viral pathogens), consist of pattern recognition receptors (PRRs), adaptor protein (apoptosis-associated speck-like protein containing a CARD; ASC), and pro-caspase-1. Recent studies have reported several PRRs for viruses including herpesviridae [5,6,7,8,9,10].

In this study, we found that EBV infection induced caspase-1-dependent IL-1β secretion in THP-1 cells (human monocyte cell line) and human primary monocytes. Of the PRRs examined, AIM2 expression was upregulated during EBV infection, and the activated caspase-1 level was downregulated in AIM2 knockdown THP-1 cells. These findings suggested that an AIM2 inflammasome was involved in the inflammatory response of monocytes to EBV infection.

## Materials and methods

### Cells

THP-1 cells: human acute monocytic leukemia cell line (JCRB Cell Bank, Ibaraki, Japan); BJAB cells: Burkitt lymphoma cell line; Jurkat cells: Acute T lymphoblastic leukemia cell line; and B95-8 cells: EBV-producing marmoset B-cell line were grown in RPMI-1640 medium supplemented with 10% FCS, 1% GlutaMAX (Invitrogen, Carlsbad, CA), and 1% penicillin-streptomycin. The human CD14-positive monocytes from peripheral blood were purchased from PromoCell (Heidelberg, Germany) and were grown in the prescribed medium. AGS-EBV-GFP cells, which are human gastric adenocarcinoma cells producing the recombinant EBV with green fluorescent protein (GFP), were grown in F-12 HAM’s medium supplemented with 10% FCS, 1% penicillin-streptomycin, and 420 μg/mL G418 [11].

### Antibodies and reagents

LPS (*Escherichia coli* 0111:B4) was from Sigma-Aldrich (St. Louis, MO). Caspase-1 Inhibitor VI was from EMD Millipore (Billerica, MA) and was used at 10 μM. Antibodies used for flow cytometry and western blotting are listed in Table 1.

**Table 1:** Antibodies used in this study.

Antibody	Cat#	Source
Rabbit anti-human caspase-1	AHZ0082	Invitrogen
Rabbit anti-human AIM2	14–6008	eBioscience
Mouse anti-human NLRP3	ALX-808-818	Enzo Life Sciences
Mouse anti-human β actin	#3700	Cell Signaling Technology
Mouse anti-human CD21	#354911	BioLegend
Isotype (mouse IgG1)	#400125	BioLegend
Mouse anti-human CD35	#333405	BioLegend
Isotype (mouse IgG1)	#400113	BioLegend
Mouse anti-human HLA-DR	#A07793	Beckmann Coulter
Isotype (mouse IgG1)	#A07798	Beckmann Coulter

### Flow cytometry

To analyze their surface antigens, THP-1 cells were washed with flow cytometry buffer (PBS with 2% FBS) and incubated with anti-CD35, CD21 or HLA-DR antibodies or with isotype control antibodies diluted in 100 μl buffer (PBS with 0.5% BSA and 0.1% sodium azide) for 30 minutes. Samples were washed twice and suspended in flow cytometry buffer. To analyze AGS-EBV-GFP infected cells, cells were collected after infection, washed with flow cytometry buffer and fixed with 1% paraformaldehyde. Prepared samples were analyzed by flow cytometry, using a FACSCalibur (BD Biosciences, San Jose, CA), and data were analyzed using FlowJo version 9.9.4 (FLOWJOE, LLC, Ashland, OR).

### EBV infection and virus titration

EBV was obtained from the 10-day-old cell-free supernatant of AGS-EBV-GFP or B95-8 cells. The supernatant was filtered (0.45 μm) and concentrated by centrifugation at 16,000 *g* for 90 minutes at 4°C. The virus stock was stored at -80°C. To titrate AGS-EBV-GFP virus, 100 μL of virus stock was incubated with 1×10^6^ BJAB cells for 2 hours at 37°C, after which 1 mL of media was added and the suspension was cultured in a single well of a 12-well culture plate at 37°C. After 48 hours of incubation, the proportion of GFP-positive cells was quantified using flow cytometry. The following equation was used for titration of the virus stock [12]:

$$\text{GBU}/\text{mL}=\;-\text{In }\left(1-\frac{n\text{GFP}}{n\text{BJAB}}\right)\;\times n\text{total}\;\times \text{dilution factor }$$

Where:

GBU is the Green BJAB Unit
*n*GFP is the number of GFP positive cells quantified by flow cytometry
*n*BJAB is the total number of BJAB cells quantified by flow cytometry
*n*total is the overall total number of cells

Using this equation, the GBU of our virus stock was 6.0×10^6^ /mL.

EBV infections were performed by suspending 1×10^6^ cells in 100 μL of the virus stock, prepared as described above. Cells were incubated for 2 hours at 37°C, and were then added to RPMI medium and cultured until collection of cell lysates or supernatant samples. At 48 hours post incubation, EBV infection was confirmed by confocal microscopy, using a TIE-A1R (Nikon, Tokyo, Japan), and the proportion of GFP-positive cells was quantified using flow cytometry. To determine the minimal viral titer for infection, THP-1 and BJAB cells were also incubated with diluted virus stock (6.0×10^5^ or 6.0×10^4^ GBU/mL).

To obtain virion-free supernatant, the AGS-EBV-GFP cell-free supernatant was filtered twice through 0.1 μm filters and was then concentrated by centrifugation at 16,000 *g* for 90 minutes at 4°C. BJAB cells were incubated with virion-free supernatant, and the absence of GFP-positive cells was confirmed after 48 hours of incubation.

### RNA isolation and RT-PCR assays

Total RNA was extracted from cells using the RNeasy mini kit (Qiagen, Hilden, Germany), following the manufacturer’s instructions. Contaminating DNA was removed by on-column DNase digestion using the RNase-free DNase set (Qiagen).

To evaluate EBV infection status, the pattern of EBV infection was determined by quantifying the expression of six latent (EBNA1, EBNA2, LMP1, LMP2, EBER1, and BARTs) and two lytic (BZLF1 and glycoprotein 350/220) EBV genes by one-step RT-PCR, using QuantiFast Multiplex RT-PCR Kits (Qiagen) as described previously [13,14]. The stably-expressed housekeeping gene, beta-2-microglobulin, was used as an endogenous control and reference gene for relative quantification.

Inflammasome-related mRNA expression was quantified by real-time PCR using SYBR Premix Ex Taq II (Takara, Kusatsu, Japan). 18S ribosomal RNA was used as an endogenous control and reference gene for relative quantification. cDNA was synthesized using the SuperScript III First-Strand System for RT-PCR (Invitrogen). The primers are listed in Table 2.

**Table 2:** Primers used in this study.

name	primers (forward / reverse)
IL-1β	5'TTCTTCGACACATGGGATAACG3'
	5'TGGAGAACACCACTTGTTGCT3'
18s	5'CACGGCCGGTACAGTGAAAC3'
	5'CCCGTCGGCATGTATTAGCT3'
AIM2	5'ATGCAGCAGGACTCATTTCA3'
	5'CGTCTTCAGGAGGAGAAGGA3'
IFI16	5′ACAAACCCGAGAAACAATGACC3′
	5′GCATCTGAGGAGTCCGAAGA3'
caspase-1	5'AATACTGTCAAATTCTTCATTGCAGATAAT3'
	5'AAGTCGGCAGAGATTTATCCAATAA3'
NLRP3	5'CACCTTGATATGGTGCAGTGTGT3'
	5'CCCGGCAAAAACTGGAAGT3'

### Real-time PCR assay of EBV DNA

Viral DNA was extracted from THP-1 or BJAB cells using QIAamp DNA blood kits (Qiagen) after cell counting. The real-time PCR assay was performed in a total reaction mixture containing 5 μl of DNA extract, 12.5 μl of QuantiTect multiplex PCR master mix (QIAGEN), forward and reverse primer, and probe as previously described [15]. The number of viral DNA copies was calculated from the standard curves and expressed as copies per cell.

### ELISA assay

Cell-free supernatants were collected at each time point after infection or treatment with RPMI, or LPS (10 ng/ml). The supernatants were analyzed for the level of IL-1β and IL-18 using Quantikine ELISA (R&D systems, Minneapolis, MN), following the manufacturer’s instructions. Briefly, culture supernatants and standards were incubated in the antibody-precoated wells for 2 hours. The wells were washed three times with washing buffer and were then incubated with conjugate for 1 hour. After three washes, the wells were incubated with substrate solution for 20 minutes protected from light. The reaction was terminated by adding stop solution. After 5 minutes, readings were taken at 450 nm using a microplate reader. Readings were calculated using a standard curve.

### Western blot analysis

Caspase-1 in the cell lysate and supernatant and PRR proteins in the cell lysates were analyzed by western blotting. The cells were washed with PBS and lysed in Laemmli sample buffer (Bio-Rad, Hercules, CA) containing β-mercaptoethanol (Katayama Chemical Industries, Osaka, Japan). Proteins were separated by SDS-PAGE through 4–15% polyacrylamide, and transferred onto a nitrocellulose membrane. Membranes were pretreated in Tris-buffered saline-Tween 20 with 5% dry milk for 1 hour, then incubated overnight at 4°C with primary antibodies diluted in the Can Get Signal immunoreaction enhancer solution 1 (Toyobo, Osaka, Japan). Membranes were washed three times with Tris-buffered saline-Tween 20 and incubated with the secondary antibodies diluted in the Can Get Signal immunoreaction enhancer solution 2 (Toyobo) for 1 hour at room temperature or overnight at 4°C.

### RNA interference assay

Human small interfering RNA (siRNAs) for AIM2 (M011951-00-0005, siGENOME Human AIM2 siRNA SMARTpool), and nontargeting control (D001206-14-05, siGENOME Non-Targeting siRNA Pool #2) were obtained from GE Dharmacon (Lafayette, CO). Nucleofection was performed using the SG Cell Line 4D-Nucleofector X Kit (Lonza, Basel, Switzerland), as previously described [16]. For transfection, 2×10^6^ THP cells were suspended in 100 μL of Nucleofector Solution with 300 nM si-RNA, then transferred to a nucleofection cuvette. Electroporation was performed with a 4D-Nucleofector (Lonza), using the Y-001 program. Cells were transferred to 6-well plates with pre-warmed medium and cultured for 72 hours, then incubated with EBV.

### Statistical analysis

Statistical significance was tested by Student’s t test or by one-way ANOVA with Tukey’s post hoc test using SPSS version 23.0 (IBM, Chicago, IL, USA). A *p*-value of less than 0.05 was considered statistically significant.

## Results

### EBV infects THP-1 cells and induces an abortive lytic infection

Prior to analyzing EBV-induced inflammasome activation in monocytes, we determined whether EBV could infect a monocyte cell line (THP-1). THP-1 and BJAB cells were incubated with various concentrations of a cell-free AGS-EBV-GFP supernatant (6.0×10^6^, 6.0×10^5^ or 6.0×10^4^ GBU/mL).

At 48 hours post incubation with 6.0×10^6^ GBU/mL of the AGS-EBV-GFP cell supernatant, almost half of the BJAB cells were GFP positive (44.7%) by FACS analysis, whereas no GFP-positive cells were observed following incubation with a virion-free supernatant (filtered). When incubated with 6.0×10^4^ GBU/mL, 1.96% of the cells were GFP-positive. Regarding THP-1 cells, a few GFP-positive cells (1.73%) were observed following incubation with 6.0×10^6^ GBU/mL. GFP-positive cells were still detected following incubation with 6.0×10^5^ GBU/mL (1.07%), while no significant GFP positive cells were observed following incubation with 6.0×10^4^ GBU/mL. Jurkat cells were also incubated with a cell-free AGS-EBV-GFP supernatant (6.0×10^6^ GBU/mL) and a few GFP-positive cells were also observed (2.2%) (S1 Fig).

Surface antigens known to be EBV receptors were analyzed in THP-1 cells using FACS. Although CD21 was absent, CD35 and HLA-DR were expressed in THP-1 cells (S1 Fig).

**Fig 1:**
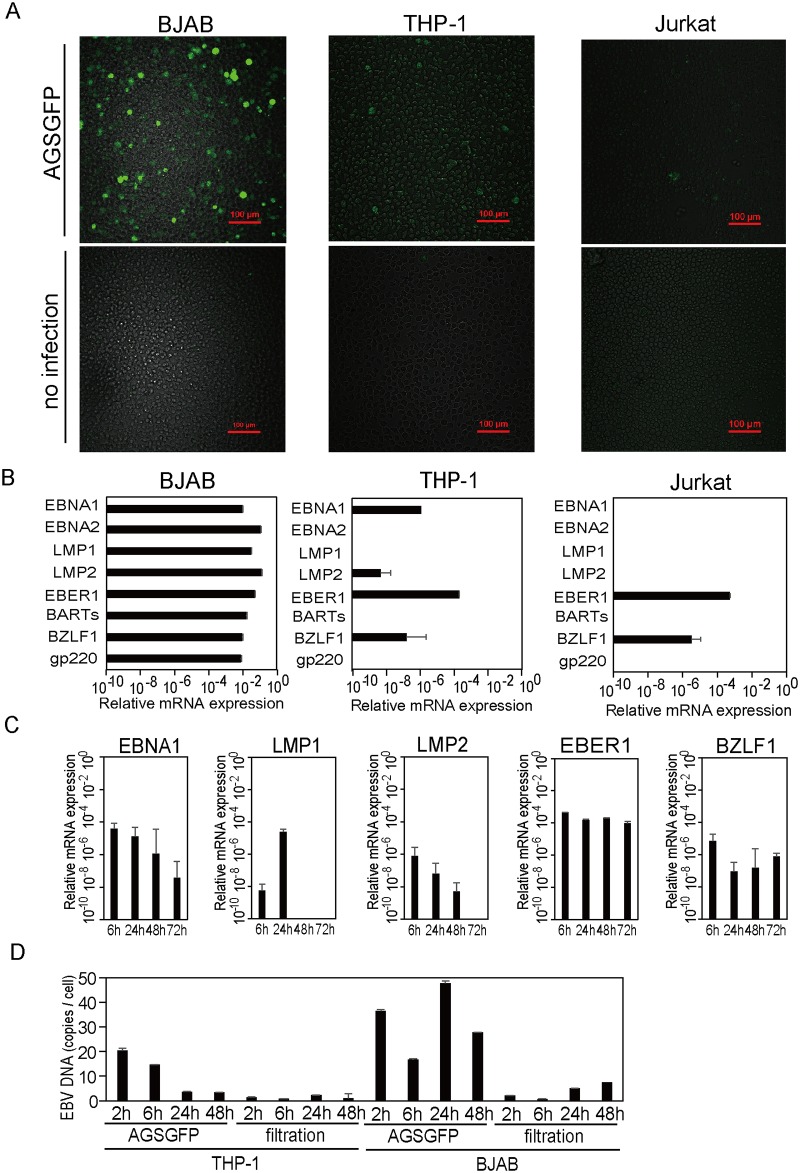
EBV infection in THP-1, Jurkat, and BJAB cells. (A) Confocal microscopic images of BJAB, THP-1, and Jurkat cells 48 hours after incubation with an AGS-EBV-GFP cell supernatant (AGSGFP) or RPMI (no infection). (B) EBV gene expression in BJAB, THP-1, and Jurkat cells 48 hours after incubation with an AGS-EBV-GFP supernatant. EBV gene expression was quantified relative to beta-2-microglobulin expression. (C) EBV gene expression in THP-1 cells over time, following incubation with an AGS-EBV-GFP supernatant. (D) EBV DNA copies per cell in THP-1 and BJAB cells over time, following incubation with 6.0×10^6^ GBU/mL of an AGS-EBV-GFP supernatant (AGSGFP) or a virion-free supernatant (filtration). In (B, C, D) the data represent one experiment with triplicate samples. The error bars represent S.E.

AGS-EBV-GFP infected BJAB, THP-1 and Jurkat cells were also detected using confocal microscopy at 48 hours post infection (Fig 1A). When THP-1 cells incubated with a cell-free AGS-EBV-GFP supernatant were cultured for longer periods in RPMI medium with G418 in order to select cells infected with recombinant EBV with the neomycin resistant gene, GFP-positive THP-1 cells decreased by 72 hours post infection and disappeared within a week.

The pattern of EBV infection was determined by analysis of the mRNA expression of six latent and two lytic EBV genes. At 48 hours post infection, the expression of all eight EBV genes was detected in BJAB cells, that of four genes (LMP2, EBNA1, EBER1, BZLF1) was detected in THP-1 cells, and that of two genes (EBER1 and BZLF1) was detected in Jurkat cells (Fig 1B). In THP-1 cells, the expression of four latent genes (LMP1, LMP2, EBNA1, EBER1) and one lytic gene (BZLF1) was detected at 6 hours post infection; most latent gene expression levels decreased over time (Fig 1C).

To investigate viral DNA replication in EBV infected cells, EBV DNA copies per cell in THP-1 and BJAB cells were quantified using real time PCR. EBV DNA loads decreased to background levels over time after incubation in THP-1 cells. In contrast, EBV DNA levels were sustained and were detected in BJAB cells 24 hours post infection (Fig 1D).

### EBV activates caspase-1 and induces IL-1β production in THP-1 cells

**Fig 2:**
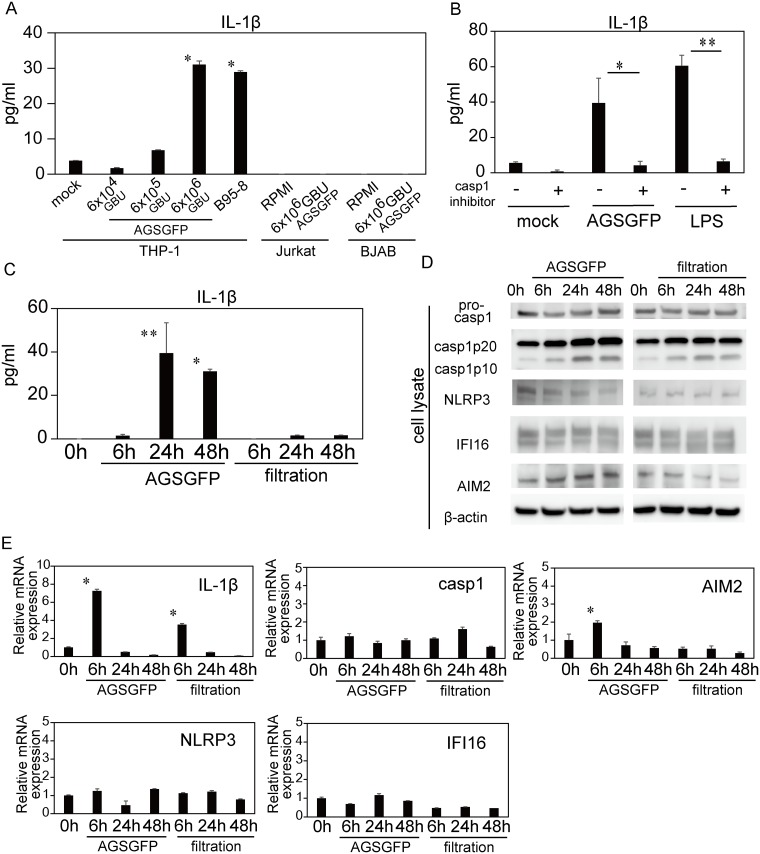
Inflammasome-related protein and gene expression of THP-1 cells after EBV infection. (A) IL-1β concentration in the supernatant of THP-1 cells, Jurkat cells, or BJAB cells at 48 hours after incubation with RPMI (mock), AGS-EBV-GFP cell supernatant (AGSGFP: 6.0×10^6^, 6.0×10^5^ and 6.0×10^4^ GBU/mL), or B95-8 cell supernatant (B95-8). The data represent two experiments each run with duplicate samples. The error bars represent S.E. The asterisk (*) indicates p < 0.001 based on comparison to the 0 h time-point by one-way ANOVA with Tukey’s post hoc test. (B) IL-1β concentration in the supernatant of THP-1 cells, pretreated with or without caspase-1 inhibitor, at 24 hours after incubation with RPMI, AGSGFP (6.0×10^6^ GBU/mL) or LPS (10 ng/mL). The data represent two experiments each run with duplicate samples. The error bars represent S.E. The single asterisk (*) indicates p = 0.003 and the double asterisk (**) indicates p < 0.001 by one-way ANOVA with Tukey’s post hoc test. (C) IL-1β concentration in the supernatant of THP-1 cells over time, following incubation with 6.0×10^6^ GBU/mL of AGSGFP supernatant or virion-free supernatant (filtration). The data represent one experiment with triplicate samples. The error bars represent S.E. The single asterisk (*) indicates p = 0.041 and the double asterisk (**) indicates p = 0.003 based on comparison to the 0 h time-point by one-way ANOVA with Tukey’s post hoc test. (D) Immunoblot analysis of caspase-1, NLRP3, IFI16, and AIM2 in THP-1 cell lysates over time, following incubation with 6.0×10^6^ GBU/mL of AGSGFP supernatant or virion-free supernatant (filtration). (E) The mRNA expression of inflammasome-related genes (IL-1β, caspase-1, AIM2, NLRP3 and IF116) in THP-1 cells after incubation with 6.0×10^6^ GBU/mL of AGSGFP cell supernatant or virion-free supernatant (filtration) was measured using RT-PCR. The data are expressed as fold changes compared to the 0 h time-point. The data represent one experiment with triplicate samples. The error bars represent S.E. The asterisk (*) indicates p < 0.001 based on comparison to the 0 h time-point by one-way ANOVA with Tukey’s post hoc test.

The supernatant levels of IL-1β significantly increased in THP-1 cells incubated with an AGS-EBV-GFP (6.0×10^6^ GBU/mL) or a concentrated B95-8 supernatant for 48 hours compared with mock infected cells. IL-1β did not significantly increase in THP-1 cells incubated with 6.0×10^5^ or 6.0×10^4^ GBU/mL. In contrast to THP-1 cells, the supernatant levels of IL-1β did not increase in similarly treated BJAB or Jurkat cells (Fig 2A). To determine whether the IL-1β secretion was caspase-1-dependent, THP-1 cells pretreated with or without caspase-1 inhibitor were incubated with RPMI medium, AGS-EBV-GFP (6.0×10^6^ GBU/mL) or LPS (10 ng/ml) and were cultured for 24 hours. IL-1β release from EBV-infected or LPS-treated THP-1 cells was reduced by caspase-1 inhibitor treatment (Fig 2B). Cell viability did not differ between the samples with or without caspase-1 treatment (no treatment vs. caspase-1 inhibitor treatment: 99.4% vs. 99.2% in mock; 99.5% vs. 99.1% in AGSGFP; 98.4% vs. 98.3% in LPS).

The THP-1 cell supernatant levels of IL-1β had increased at 24 hours after incubation with AGS-EBV-GFP, but had not increased after incubation with virion-free supernatant (Fig 2C). Western blotting indicated that the protein levels of cleaved caspase-1 (p20 and p10) in THP-1 cell lysates also increased with time after incubation with the AGS-EBV-GFP supernatant (Fig 2D). Of the PRRs, the protein levels of AIM2 increased over time only with viral infection, whereas the levels of IFI16 and NLRP3 showed no remarkable change. Cleaved caspase-1 (p20 and p10) levels also increased with time after incubation with the virion-free supernatant, although none of the PRR proteins showed any increase under this condition (Fig 2D).

Analysis of mRNA expression demonstrated that IL-1β mRNA showed a 7.2-fold increase at 6 hours post incubation with AGS-EBV-GFP compared to 0 hour. IL-1β mRNA also showed a 3.5-fold increase at 6 hours post AGS-EBV-GFP incubation compared with 6 hours post incubation with virion-free supernatant. AIM2 mRNA increased with EBV infection, but did not increase following incubation with virion-free supernatant. NLRP3, IFI16, and caspase-1 mRNA did not show a remarkable change under either condition (Fig 2E).

### EBV infects primary human monocyte cells and induces IL-1β production

**Fig 3:**
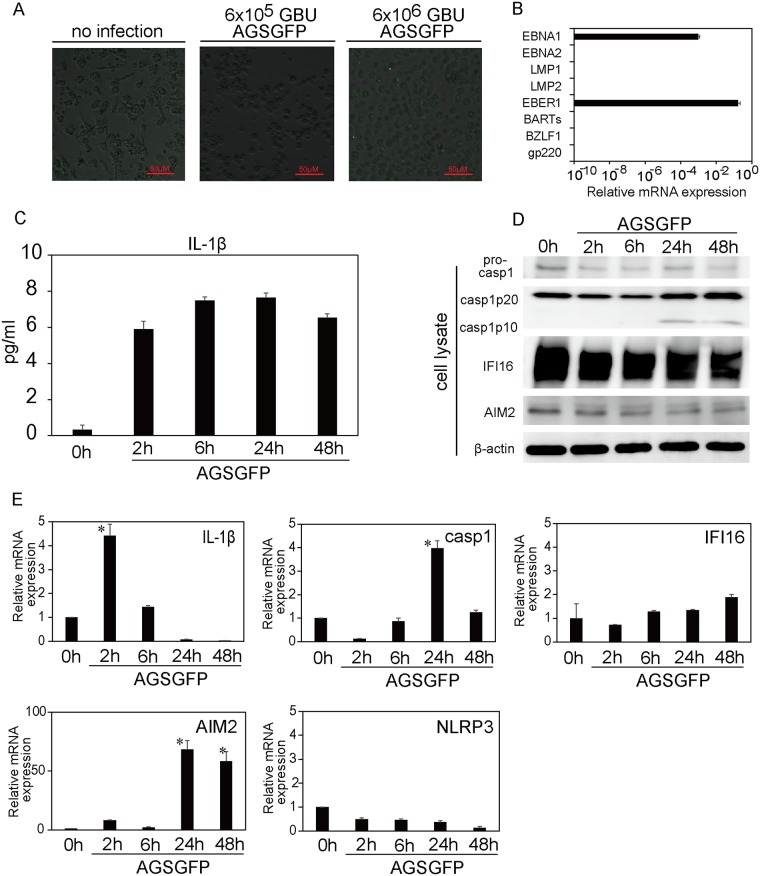
EBV infection and inflammasome activation in human monocytes. (A) Confocal microscopic images of human monocytes 48 h after incubation with RPMI medium (no infection) or an AGS-EBV-GFP cell supernatant (AGSGFP: 6.0×10^6^ or 6.0×10^5^ GBU/mL). (B) EBV gene expression of human monocytes at 48 h after incubation with 6.0×10^6^ GBU/mL of an AGSGFP cell supernatant. EBV gene expression was quantified relative to beta-2-microglobulin expression. The data represent one experiment with triplicate samples. The error bars represent S.E. (C) IL-1β concentration in the supernatant of human monocytes over time, following incubation with 6.0×10^6^ GBU/mL of an AGSGFP cell supernatant. The data represent one experiment with duplicate samples. The error bars represent S.E. (D) Immunoblot analysis of caspase-1, IFI16, and AIM2 protein expression in human primary monocyte lysates over time, following incubation with 6.0×10^6^ GBU/mL of an AGSGFP cell supernatant. (E) Inflammasome-related gene (IL-1β, caspase-1, AIM2, NLRP3 and IFI16) expression of human monocytes over time, following incubation with 6.0×10^6^ GBU/mL of an AGSGFP cell supernatant, was measured using RT-PCR. The data are expressed as fold changes compared to the 0-hour time-point. The data represent one experiment with triplicate samples. The error bars represent S.E. The asterisk (*) indicates p < 0.001 based on comparison to the 0-hour time-point by one-way ANOVA with Tukey’s post hoc test.

Primary human monocytes were incubated with a concentrated AGS-EBV-GFP supernatant (6.0×10^6^ or 6.0×10^5^ GBU/mL) for 2 hours and were then cultured for various periods of time. A few cells were GFP-positive at 48 hours after incubation with 6.0×10^6^ GBU/mL of AGS-EBV-GFP (Fig 3A). Analysis of EBV gene expression at this time point showed the elevation of two latent genes (Fig 3B). The IL-1β level in the supernatant was elevated with EBV infection (Fig 3C). In cell lysates, cleaved caspase-1 was observed at 24 and 48 hours after EBV infection, whereas no remarkable change in the IFI16 or AIM2 protein level was observed (Fig 3D). The mRNA expression of IL-1β increased at 2 hours post EBV incubation and caspase-1 mRNA increased at 24 hours post incubation (Fig 3E). AIM2 mRNA significantly increased at 24 hours after EBV incubation, whereas no increase was observed in IFI16 or NLRP3 mRNA levels (Fig 3E).

### AIM2 knockdown in THP-1 cells downregulated EBV-induced caspase-1 activation

**Fig 4:**
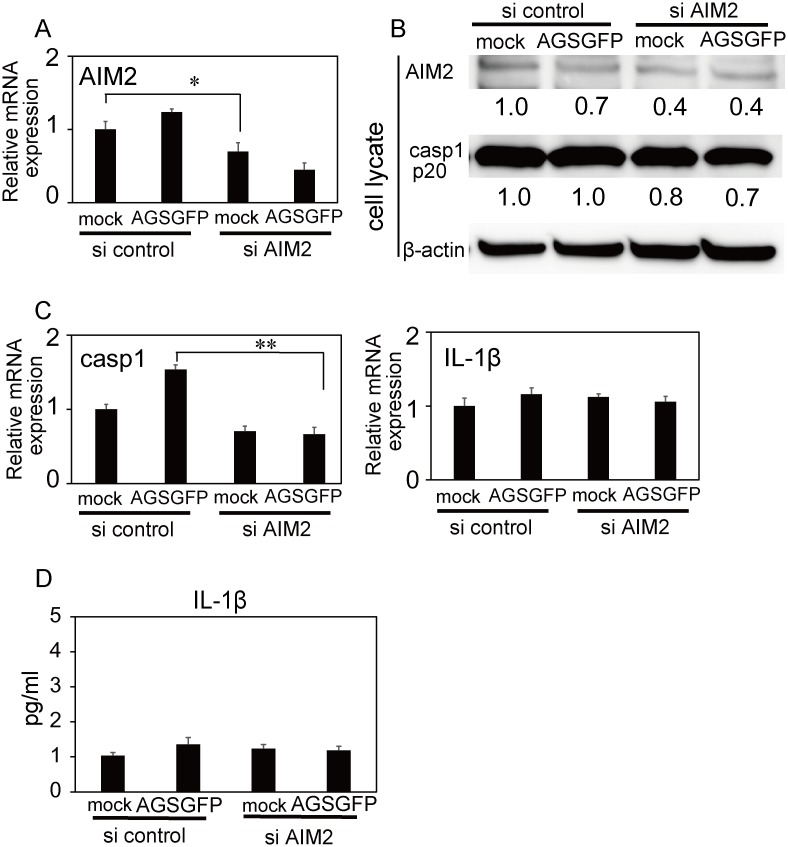
Inflammasome activation in AIM2 knockdown THP-1 cells after EBV infection. The following analyses were performed at 24 hours after incubation with RPMI (mock) or AGS-EBV-GFP cell supernatant (AGSGFP). (A) AIM2 mRNA expression of siRNA-treated THP-1 cells. The asterisk (*) indicates p = 0.02 (B) Immunoblot analysis of siRNA-treated THP-1 cells. β-actin was blotted as a loading control. (C) mRNA expression of caspase-1 and IL-1β in siRNA-treated THP-1 cells. The double asterisk (**) indicates p<0.001 by one-way ANOVA with Tukey’s post hoc test. (D) IL-1β concentration in siRNA-treated THP-1 cells. (A-D) The data are expressed as fold changes compared to si-control-treated THP-1 cells incubated with RPMI (mock). (A, C, D) The data represent one experiment with triplicate samples. The error bars represent S.E.

To determine whether AIM2 was involved in the inflammasome response in EBV infected THP-1 cells, we investigated the effect of AIM2 knockdown by RNA interference in THP-1 cells. Real-time RT-PCR and immunoblot analysis revealed that siAIM2 treatment reduced both the mRNA and protein levels of AIM2 (Fig 4A and 4B). The increase in caspase-1 mRNA following EBV infection that was observed in si control cells was not observed in siAIM2-treated THP-1 cells (Fig 4C). However, there was no difference in the cell lysate level of cleaved caspase-1 or the supernatant level of IL-1β between si control and si AIM2 cells following EBV infection (Fig 4B and 4D).

## Discussion

Human B lymphocytes and epithelial cells are major targets of EBV, but several studies have also shown that monocytes or monocyte cell lines are vulnerable to EBV infection [1,2]. However, the immunological response of monocytes to EBV infection has not been elucidated. In this study, we demonstrated that EBV could infect a small proportion of THP-1 cells and primary human monocytes, and induce inflammasome activation.

The mechanism of EBV infection of monocytes had been unclear, especially given that they lack CD21, the primary receptor for EBV in other cells. However, Ogembo et al. demonstrated that EBV infection could occur via CD35 and HLA-DR in CD21-deficient cells [17], and these molecules have been thought to be the receptor for EBV in THP-1 cells and primary human monocytes. We confirmed the expression of CD35 and HLA-DR and the absence of CD21 in THP-1 cells using flow cytometry; the surface expression levels of CD35 and HLA-DR that we detected in THP-1 cells were equivalent to, or higher than those of previous reports [18,19]. The expression of EBNA1, EBER1, and the immediate-early lytic gene BZLF1 was detected in THP-1 cells. On the other hand, expression of the latent genes LMP1 and LMP2 was transient, and the late lytic gene gp220 was not detected. Savard et al. reported the release of infectious EBV viral particles from EBV-infected primary human monocytes and the detection of BZLF1 by western blotting [2]. In our study, BZLF1 gene expression was elevated in EBV-infected THP-1 cells, but EBV DNA replication was not observed. Our finding may suggest that EBV could infect THP-1 cells, but that it then entered an abortive lytic cycle. EBV-infected THP-1 cells failed to immortalize, and disappeared within a week. One possible mechanism for this disappearance may be pyroptosis induced by activated caspase-1 [20]. *In vivo*, monocytes infected with EBV from HIV patients has been reported [21], but whether monocytes become infected by EBV in immunocompetent hosts remains unknown.

Ansari et al. demonstrated IFI-16 inflammasome activation in B and epithelial cells latently infected with EBV [6], but inflammasome activation with primary EBV infection has, thus far, not been reported. We confirmed that EBV infects BJAB cells efficiently, but release of IL-1β was not observed, suggesting that primary EBV infection of B cells does not induce inflammasome activation.

Monocytes play a central role in the innate immune response, and several studies have reported interactions between monocytes and EBV [22,23]. In this study, we aimed to clarify whether inflammasome activation is induced in monocytes during primary EBV infection. The inflammasome is composed of sensor proteins, adaptor proteins, and pro-caspase-1. When the inflammasome is assembled in cells of the innate immune system, it induces caspase-1 activation, followed by production of IL-1β or IL-18; IL-1β and IL-18 contribute to host defense during infection [24,25]. We found that THP-1 cells and human primary monocytes release IL-1β when incubated with EBV-containing supernatant. IL-1β mRNA was elevated at 6 hours post -incubation with AGSGFP, and the IL-1β levels of the supernatant were elevated at 24 and 48 hours post incubation. Before IL-1β proteins are released from cells, activated caspase-1 is required to process pro IL-1β and this requirement may explain the time lag between IL-1β mRNA expression and the release of IL-1β in this study. The protein level of AIM2, the cytoplasmic sensor molecule, was elevated over time after infection of THP-1 cells despite the fact that AIM2 mRNA increased only at 6 hours post EBV incubation and subsequently decreased. Sustainment of the protein level of PRRs after peak expression of their mRNAs was also observed in another study [26]. Furthermore, the AIM2 mRNA level was significantly increased in primary human monocytes after EBV infection.

Other studies have demonstrated involvement of the IFI16 inflammasome in herpesviridae infections, such as infections of Kaposi's sarcoma-associated herpesvirus (KSHV) and herpes simplex virus (HSV)-1 [27,28]. After entering the cell, the herpesviridae nucleocapsid is transferred to the nucleus, where viral DNA is released. Presumably, therefore, herpesviridae is sensed by IFI16, which localizes to the nucleus. However, IFI16-independent IL-1β production in KSHV infection has been reported [29], and it has also been suggested that HSV-1 DNA can be sensed in the cytosol via proteasomal degradation [30]. Furthermore, involvement of the NLRP3 inflammasome was shown in HSV-1 [28] and varicella-zoster virus [10] infections. On the other hand, an AIM2 inflammasome-dependent IL-1β response to murine cytomegalovirus infection was reported in mouse macrophages [5]. AIM2 is the cytoplasmic sensor that recognizes dsDNA and assembles the inflammasome.

We evaluated EBV DNA load in THP-1 and BJAB cells after EBV infection using real-time PCR. EBV DNA loads decreased over time in THP-1 cells. The failure of EBV DNA replication in THP-1 cells may suggests deficient entry of EBV into the nucleus resulting in insufficient sensing of viral DNA by IFI16. On the other hand, presence of viral DNA in cytoplasm was not evaluated in this study, and the cellular location of the viral DNA which should be explored in future investigations. When incubated with a virion-free supernatant of AGS-EBV-GFP cells, IL-1β was not secreted by THP-1 cells despite an observed small increase in IL-1β mRNA. Although the level of cleaved caspase-1 increased, no upregulation of AIM2, NLRP3, or IFI16 was observed. These findings may suggest a deficient inflammasome response to some cell fragment contained in the supernatant; however, this phenomenon was not further investigated in this study.

We confirmed, using real-time RT-PCR, that AIM2 knockdown in THP-1 cells attenuated EBV-induced caspase-1 activation. However, the effect of AIM2 knockdown on caspase-1 activation could not be shown at the protein level. It is possible that nucleofection efficacy or cell viability were insufficient to evaluate the inflammasome response. Another possibility is that there might be other mechanisms for caspase-1 activation in EBV-treated THP-1 cells.

Although our data imply AIM2 inflammasome involvement in EBV infected THP-1 cells and human primary monocytes, further investigation of this point is essential. A limitation of our study is the lack of proof of the existence of the AIM2 inflammasome in the cytoplasm. Immunoprecipitation with ASC might facilitate future investigations of the existence of this inflammasome complex.

Differentiated THP-1 cells have often been used in inflammasome studies [9,31,32]. 12-*O*-tetradecanonylphobol-13-acetate (TPA) treatment induces THP-1 monocytes to differentiate into THP-1 macrophages [33]. Prior to this study, we tested THP-1 macrophages pretreated with a variety of concentrations of TPA. We could not, however, evaluate inflammasome activation because the levels of IL-1β secretion were consistently elevated in differentiated THP-1 macrophages, regardless of LPS or EBV stimulation. TPA induces the release of endogenous ATP [34], and activation of caspase-1 by ATP may induce hypersensitivity.

It has been suggested that EBV infection may be controlled primarily by adaptive immunity, but little is known about innate immunity or inflammasome activation in response to primary EBV infection. Recently, Arbore et al. demonstrated that NLRP3 inflammasome activity plays an important role in human adaptive T helper type 1 response to viral infection [35]. In this study, the supernatant levels of IL-18 were increased in Jurkat cells when incubated with cell-free EBV (data not shown). IL-18 is another pro-inflammatory cytokine that is closely associated with inflammasome activation. IL-18 promotes the production of IFN*γ* from T and NK cells [36]. Details of inflammasome activation of EBV-infected T cells were not examined in this study, but it might be possible that AIM2 or other inflammasome activation is associated not only with innate immune responses, but also with adaptive immunity against EBV infection.

## Supporting information

S1 Fig EBV infection of BJAB, THP-1, and Jurkat cells and the expression of the surface antigens in THP-1 cells. GFP positive cells were analyzed by flow cytometry at 48 hours post incubation with RPMI (no infection), AGS-EBV-GFP cell supernatant (AGSGFP), or virion-free supernatant (filtration) in BJAB (A), THP-1 (B), and Jurkat cells (C). The expression of surface antigens (CD21, CD35, and HLA-DR) in THP-1 cells (D).
(TIF)
