# Supplementary figures and images for: Epstein-Barr virus infection-induced inflammasome activation in human monocytes
Source: PLoS One. 2017 Apr 3;12(4):e0175053. doi: 10.1371/journal.pone.0175053 (PMC5378412; doi:10.1371/journal.pone.0175053)

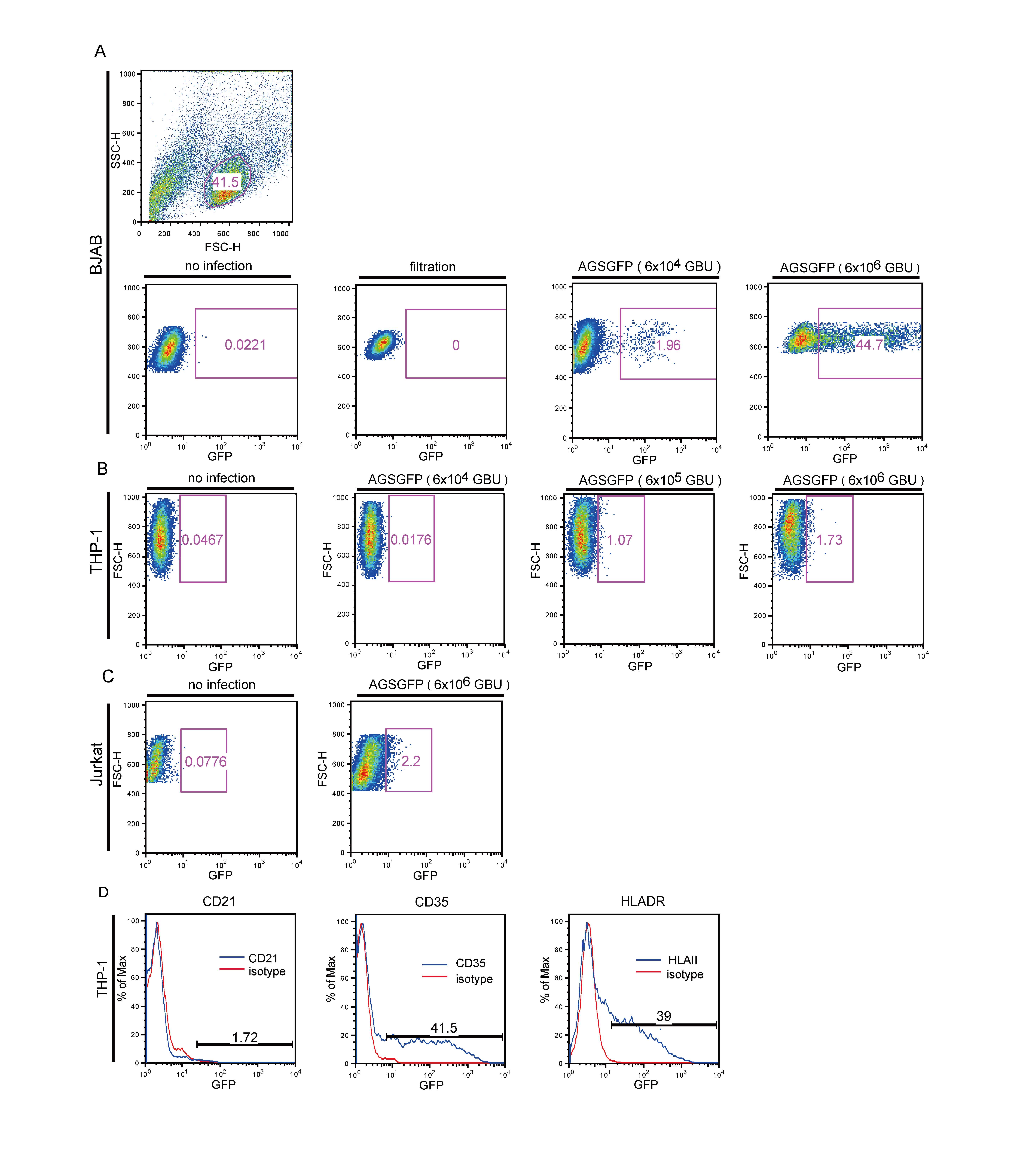

Supplement: S1 Fig — GFP positive cells were analyzed by flow cytometry at 48 hours post incubation with RPMI (no infection), AGS-EBV-GFP cell supernatant (AGSGFP), or virion-free supernatant (filtration) in BJAB (A), THP-1 (B), and Jurkat cells (C). The expression of surface antigens (CD21, CD35, and HLA-DR) in THP-1 cells (D). (TIF) [file pone.0175053.s001.tif]
